# Effects of menthol on thermoregulatory responses after exercise-induced hyperthermia

**DOI:** 10.1186/2046-7648-4-S1-A6

**Published:** 2015-09-14

**Authors:** Young-Joon Jang, Jung-Hyun Kim, Joo-Young Lee

**Affiliations:** 1 COM:FORT Laboratory, College of Human Ecology, Seoul National University, Seoul, Korea; 2 National Personal Protective Technology Laboratory (NPPTL), NIOSH/CDC, Pittsburgh, PA, USA

## Introduction

Menthol is a chemical compound stimulating TRPM8 which is activated in relatively cool environment below 28 °C [1] and induces cooling sensation on human skin [2]. Many researchers have explored menthol's thermo-physiological influences on humans. Lee and colleagues [3] found that rectal temperature increased faster and total sweat rate was lower than control when a 0.8 % menthol solution was applied on the upper body. Most researches have focused on menthol application during or before heat exposure, but in the practical situation menthol products are used for relieving thermal discomfort or muscular fatigue after intensive exercise. Therefore, the effects of topical menthol application on physiological responses, including sweat secretion after exercise-induced hyperthermia were investigated in the present study. We hypothesized that menthol application during recovery after exercise would relieve thermal sensation without increasing deep body temperature.

## Methods

Ten male subjects participated in this study. The exercise protocol consisted of 15 min rest on a cycle ergometer at air temperature (T_a_) of 23 °C and relative humidity (RH) of 50 %, followed by cycling at T_a _45 °C with 50 %RH until rectal temperature increased 1.5 °C higher than baseline, and a 1 h recovery period. At the 10^th ^min of the recovery, menthol or control (lanolin) cream was applied on both arms and legs, except the hands and feet. Skin temperatures, rectal temperature, active sweat gland density (ASGD) on the forearm and thigh, skin blood flow on the forearm and thigh, and subjective perceptions (thermal sensation, thermal comfort, and rate of perceived exertion) were measured. A peripheral vasomotor tone index was calculated from the difference between forearm and finger temperatures.

## Results

Rectal temperature showed a tendency to be decreased more quickly, the peripheral vasomotor tone index was smaller, and ASGD was also smaller in the menthol condition compared to the control, but the group differences were not statistically significant. Subjects felt cooler in the menthol condition than in control (P < 0.05) but the differences disappeared 30 min after the topical application.

## Conclusion

A menthol-induced peripheral vasoconstriction and lower sweating has been reported before exercise caused an increase in deep body temperature, which might not be safe for humans in heat. The present results indicate that menthol application on the extremities except the hands and feet during recovery after exercise did not increase rectal temperature but relieved thermal sensation and thermal comfort for 30 minutes. These results suggest that menthol can be effectively applied to sports therapy during recovery.
